# Significance of Anaerobes and Oral Bacteria in Community-Acquired Pneumonia

**DOI:** 10.1371/journal.pone.0063103

**Published:** 2013-05-06

**Authors:** Kei Yamasaki, Toshinori Kawanami, Kazuhiro Yatera, Kazumasa Fukuda, Shingo Noguchi, Shuya Nagata, Chinatsu Nishida, Takashi Kido, Hiroshi Ishimoto, Hatsumi Taniguchi, Hiroshi Mukae

**Affiliations:** 1 Department of Respiratory Medicine, University of Occupational and Environmental Health, Kitakyushu, Fukuoka, Japan; 2 Department of Microbiology, University of Occupational and Environmental Health, Kitakyushu, Fukuoka, Japan; Queens University Belfast, Ireland

## Abstract

**Background:**

Molecular biological modalities with better detection rates have been applied to identify the bacteria causing infectious diseases. Approximately 10–48% of bacterial pathogens causing community-acquired pneumonia are not identified using conventional cultivation methods. This study evaluated the bacteriological causes of community-acquired pneumonia using a cultivation-independent clone library analysis of the 16S ribosomal RNA gene of bronchoalveolar lavage specimens, and compared the results with those of conventional cultivation methods.

**Methods:**

Patients with community-acquired pneumonia were enrolled based on their clinical and radiological findings. Bronchoalveolar lavage specimens were collected from pulmonary pathological lesions using bronchoscopy and evaluated by both a culture-independent molecular method and conventional cultivation methods. For the culture-independent molecular method, approximately 600 base pairs of 16S ribosomal RNA genes were amplified using polymerase chain reaction with universal primers, followed by the construction of clone libraries. The nucleotide sequences of 96 clones randomly chosen for each specimen were determined, and bacterial homology was searched. Conventional cultivation methods, including anaerobic cultures, were also performed using the same specimens.

**Results:**

In addition to known common pathogens of community-acquired pneumonia [*Streptococcus pneumoniae* (18.8%), *Haemophilus influenzae* (18.8%), *Mycoplasma pneumoniae* (17.2%)], molecular analysis of specimens from 64 patients with community-acquired pneumonia showed relatively higher rates of anaerobes (15.6%) and oral bacteria (15.6%) than previous reports.

**Conclusion:**

Our findings suggest that anaerobes and oral bacteria are more frequently detected in patients with community-acquired pneumonia than previously believed. It is possible that these bacteria may play more important roles in community-acquired pneumonia.

## Introduction

Pneumonia is now the sixth and third leading cause of death in the United States and Japan, where 14.3/100,000 and 98.9/100,000 people die of the disease per year, respectively [1], [2]. Pneumonia is also a leading cause of death in the elderly (>80 years old) in both countries [1], [2]. It is estimated that the mortality of pneumonia will increase in aging population.

Having a precise understanding of the pathogens that cause pneumonia is very important to achieve prompt diagnoses and to determine proper antimicrobial treatments. However, according to previous reports, 10–48% of the causes of community-acquired pneumonia (CAP) were etiologically unknown when sputum and blood cultures were performed in combination with serological tests and tests for detecting urinary antigens [3]–[9]. In addition, relatively low incidences of anaerobes have been reported as causative bacteria (0–5.5%) [3]–[9]. It has been speculated that bacteria that are less culturable, such as anaerobes and oral bacterial flora, and they are assumed to be indigenous and tend to be ignored in sputum samples in ordinary clinical settings, may be responsible for the unknown bacteriological etiology in CAP. However, the incubation of the samples in agar plates under anaerobic conditions in clinical microbiology laboratories is not commonly performed.

Recently, the microbiota of the lower respiratory tract in patients with pulmonary infections, such as intensive care unit pneumonia [10], cystic fibrosis [11] and ventilator-associated pneumonia (VAP) [10], were studied using 16S ribosomal RNA (rRNA) gene amplification followed by clone library methods. In addition to identifying well-known causative pathogens of lower respiratory tract infections, these studies indicated the involvement of many bacteria that were previously thought to be non-pathogenic. Furthermore, information regarding bacteria obtained in the past several years using new molecular biology techniques (16S quantitative PCR followed by pyrosequencing) has highlighted the existence and possible clinical roles of the microbiota of the lower respiratory tract in patients with chronic obstructive pulmonary disease [12]–[14] and even in healthy subjects [15].

We previously reported the diagnostic utility of a clone library analysis of the 16S rRNA gene using bronchoalveolar lavage (BAL) fluid for bacteriological information in patients with pneumonia caused by *Legionella* sp. [16] and *Leptotrichia* sp. [17]. This molecular method can detect the phylotypes whose 16S rRNA gene sequences are the most similar to those of the type strains, and can determine the ratio of phylotypes (bacterial flora) in each specimen in a cultivation-free fashion.

The diagnostic utility of the culture of BAL fluid using fiberoptic bronchoscopy with higher detection rates than sputum samples in CAP patients was also reported [18].

In the present study, we performed bronchoscopy to evaluate the causative pathogens in CAP patients, and BAL specimens were analyzed by both a microfloral molecular analysis of the 16S rRNA gene and ordinary cultivation methods, in combination with serological assays and detection of urinary antigens.

## Methods

### Subjects

Sixty-four consecutive CAP patients in our university hospital and referred hospitals between April 2010 and December 2011 were enrolled in this study. Bronchoscopy was performed to evaluate the causative pathogens in the lesions of these pneumonia patients. CAP was defined according to the Infectious Diseases Society of America (IDSA)/American Thoracic Society (ATS) guidelines for diagnosing CAP in adults [19]. This study excluded patients with healthcare-associated pneumonia (HCAP) and hospital-acquired pneumonia (HAP) [20].This study was approved by the Human and Animal Ethics Review Committee of the University of Occupational and Environmental Health, Japan (No.09–118). Written informed consent was obtained from either the patients or their guardians. If the patients were under 20 years old, their parents provided written informed consent on their behalf. The following patient information was collected: age, sex, underlying diseases, clinical manifestations, and laboratory and radiological findings.

BAL specimens obtained from 30 patients with idiopathic interstitial pneumonias (IIPs) using the same methods were also evaluated as representative samples of noninfectious pulmonary diseases.

### Sample Collection

Fiberoptic bronchoscopy was performed according to the British Thoracic Society guidelines for diagnostic flexible bronchoscopy [21]. Gargling with povidone iodine solution was performed before bronchoscopy to minimize contamination by oral bacteria, and a fiberoptic bronchoscope was then introduced transorally into the trachea by passing it through the vocal cords without any contacts or aspiration to avoid oral bacterial contamination. BAL specimens were then obtained from the affected lesions using 40 ml of sterile saline. Moreover, sputum samples were also evaluated in patients with sputum production.

### Total Bacterial Cell Counts and Cell Lysis Efficiency Analyses

To provide a precise evaluation of the microbiota, we evaluated the total bacterial cell counts and the efficiency of cell lysis using epifluorescent microscopy, as previously reported [22].

### Microbiological Examination

The BAL specimens and the sputum samples were quantitatively cultivated under aerobic and anaerobic conditions as described previously [22].

Serological methods using single or paired sera were used to examine the presence of antibodies against *Mycoplasma pneumoniae* Complement Fixation Antigen (Denka Seiken, Tokyo, Japan), and *Chlamydophila psittaci* Complement Fixation Antigen (Denka Seiken, Tokyo, Japan). The level of anti- *Chlamydophila pneumoniae* antibodies was determined by the SeroCP ELISA for immunoglobulin G (IgG) and IgA (Savyon and Hain Lifescience, Nehren, Germany).

Urinary antigen tests to detect *Streptococcus pneumoniae* and *Legionella pneumophila* (Binax, Portland, ME, USA) were also performed.

### Criteria for a Conventional Etiologic Diagnosis

Bacteria were considered to be causative organisms when they were isolated from blood cultures. Any microorganism isolated from the BAL specimens was considered to be a presumptive pathogen when its concentration reached ≥10^4^ colony-forming units (CFU)/ml in the quantitative cultures [23], [24].

For serological assessment of *M. pneumoniae* and *C. pneumoniae,* a four-fold increase in antibody titer levels between the paired sera was considered to be presumptive. *L. pneumophila* and *S. pneumoniae* were considered to be presumptive agents when the urinary antigen tests were positive.

### DNA Extraction

DNA samples were extracted from the BAL specimens by vigorously shaking them with sodium dodecyl sulfate (final concentration: 3.0%) and glass beads, as reported previously [22].

### PCR Conditions

16S rRNA genes were amplified with a GeneAmp PCR system 9700 thermocycler (Applied Biosystems; Foster City, CA). The reaction mixtures containing the universal primers set [25] (E341F; 5′-CCTACGGGAGGCAGCAG-3′ and E907R; 5′-CCGTCAATTCMTTTRAGTTT-3′) and AmpliTaq Gold DNA polymerase LD (Applied Biosystems; Foster City, CA) were incubated in a thermocycler at 96°C for 5 min. This was followed by 30 cycles at 96°C for 30 s, 53°C for 30 s and 72°C for 1 min and a final elongation step at 72°C for 7 min.

### Clone Library Construction and Determination of Nucleotide Sequences

The PCR products were cloned with a TOPO TA cloning kit (Invitrogen; Carlsbad, CA) according to the manufacturer’s instructions. A total of 96 colonies were randomly selected from each clone library for sequencing analysis. The partial fragments of the cloning vectors (pCR II) containing inserted PCR products were amplified with AmpliTaq Gold DNA polymerase and a primer set (M13Forward; 5′-GTAAAACGACGGCCAG-3′ and M13Reverse; 5′-CAGGAAACAGCTATGAC-3′). After the primers and deoxyribonucleotide triphosphate were eliminated from the PCR mixture with an ExoSAP-IT (GE Health care UK Ltd.; England, UK) according to the manufacturer’s instructions, a 1-µl aliquot was used as a template for the sequencing reaction. The sequencing reactions were accomplished with primers “M13Forward” and the BigDye Terminator Cycle Sequencing Kit v3.1 (Applied Biosystems). The nucleic acid sequences were determined on a 3130*×l* Genetic Analyzer (Applied Biosystems).

### Homology Searching

Highly accurate sequences selected by the Phred quality values were compared with the 16S rRNA gene sequences of the type strains using the basic local alignment search tool (BLAST) algorithm, as described previously [22].

A phylotype sharing 97% or higher homology with the sequence of the type strain was assumed to be a presumptive species, as described previously [26], and a phylotype with a sequence sharing between 90% and 97% of the type strain was assumed to be a presumptive genus in the present study.

### Assessment of Pneumonia Severity and 30-day Mortality

The assessment of the severity of pneumonia in each patient was conducted using the pneumonia severity index (PSI) [27]. The mortality 30 days after admission was also evaluated.

## Results

### Patient Characteristics

The baseline characteristics of the patients are presented in Table 1. The average age of the 64 patients (33 males and 31 females) was 63.2 (range: 16–91) years. Forty-four patients (68.8%) had at least one comorbid illness, such as chronic pulmonary disease (20.3%), diabetes mellitus (21.9%), malignancy (14.1%), or renal disease (9.4%). None of the patients had acquired immunodeficiency syndrome or had received any organ transplant. The severity of pneumonia was evaluated using PSI scores. The mortality rates of mild, moderate, and severe cases at 30 days after admission were 0% (0/45), 11.1% (1/9), and 20% (2/10), respectively.

**Table 1:** Clinical and laboratory features of the patients with community-acquired pneumonia.

		Patients (n = 64)	IIPs (n = 30)
Age(y); mean ± SD [range]		63.2±20.7 [16–91]	61.4±20.0 [16–80]
Sex	Female; n (%)	31 (48.4)	10 (33.3)
	Male; n (%)	33 (51.6)	20 (66.7)
Comorbid diseases	Chronic pulmonary disease; n (%)	13 (20.3)	2 (6.7)
	Bronchial asthma; n (%)	3 (4.7)	1 (3.3)
	Malignancy; n (%)	9 (14.1)	5 (16.7)
	Cerebrovascular disease; n (%)	5 (7.8)	1 (3.3)
	Diabetes mellitus; n (%)	14 (21.9)	5 (16.7)
	Collagen disease; n (%)	2 (3.1)	0 (0.0)
	Cardiac disease; n (%)	5 (7.8)	7 (23.3)
	Renal disease; n (%)	6 (9.4)	1 (3.3)
	No comorbid diseases; n (%)	20 (31.3)	14 (46.7)
	Immunosuppression; n (%)	10 (15.6)	1 (3.3)
	Two or more comorbidities; n (%)	19 (29.7)	6 (20.0)
Clinical parameters	Orientation disturbance (confusion); n (%)	8 (12.5)	1 (3.3)
	Body temperature <35°C or >40°C; n (%)	3 (4.7)	0 (0.0)
	Systolic BP<90 mmHg or diastolic BP≤60 mm Hg; n (%)	1 (1.6)	1 (3.3)
	Pulse rate ≥125 beats/min; n (%)	7 (10.9)	1 (3.3)
	Respiratory rate ≥30 breaths/min; n (%)	11 (17.2)	6 (20.0)
	SpO_2_≤90%, PaO_2_≥60 Torr; n (%)	13 (20.3)	7 (23.3)
Laboratory findings	BUN ≥10.7 mmol/L; n (%)	6 (9.4)	3 (10.0)
	Na <130 mEq/ml; n (%)	1 (1.6)	0 (0.0)
	Glucose ≥13.9 mmol/L; n (%)	5 (7.8)	2 (6.7)
	Hematocrit <30%; n (%)	4 (6.3)	2 (6.7)
Radiographic findings	Involvement of one zone; n (%)	26 (40.6)	6 (20.0)
	Involvement of two or more zones, not bilateral; n (%)	6 (9.4)	5 (16.7)
	Bilateral lung involvement; n (%)	32 (50.0)	19 (63.3)
	Pleural effusion; n (%)	7 (10.9)	2 (6.7)

BUN; blood urea nitrogen, IIPs; Idiopathic interstitial pneumonias, SD; standard deviation.

### Total Bacterial Numbers Obtained with Epifluorescent Microscopic Evaluations and Cell Lysis Efficiency Analyses

The number of bacteria in each BAL specimen was counted using an epifluorescent microscopic analysis. The numbers of bacteria ranged from 1.3×10^4^ to 3.7×10^9^ (median 2.5×10^6^) cells/ml (Table 2). The efficiency of cell lysis was maintained at 80% or greater in all samples.

**Table 2:** Comparison of detected bacteria between conventional cultivation and the molecular method of bronchoalveolar lavage fluid.

		Bronchoalveolar lavage fluid				
Case No.	Age/Sex	Cell number (cells/ml)	Cultivation	The Results of clone library analysis of 16S ribosomal RNA gene		Sputum cultivation
				The predominant phylotype	(Clones/clones, %)	
1	81 M	1.5×10^6^	*Streptococcus oralis*	*Streptococcus oralis*	13/69, 18.8%	Not analyzed
2	80 M	1.5×10^8^	*Streptococcus pneumoniae, Haemophilus influenzae*	*Streptococcus pneumoniae*	65/65, 100%	Not analyzed
3	80F	4.9×10^6^	*Streptococcus pneumoniae*	*Streptococcus pneumoniae*	83/88, 94.3%	*Streptococcus pneumoniae*
4	59F	6.2×10^7^	No growth	*Prevotella tannerae*	33/77, 42.9%	Not analyzed
5	88F	1.6×10^6^	No growth	*Haemophilus influenzae*	71/77, 92.2%	Not analyzed
6	24F	9.7×10^6^	Oral bacteria^#^	*Mycoplasma pneumoniae*	83/91, 91.2%	Oral bacteria
7	74 M	1.9×10^7^	*Streptococcus pneumoniae*	*Streptococcus pneumoniae*	93/93, 100%	*Streptococcus pneumoniae*
8	29 M	9.8×10^6^	No growth	*Prevotella melaninogenica*	36/91, 39.6%	Oral bacteria
9	83 M	7.1×10^7^	*Streptococcus pneumoniae*	*Streptococcus pneumoniae*	92/92, 100%	*Streptococcus pneumoniae*
10	65 M	1.1×10^6^	No growth	*Streptococcus pneumoniae*	90/93, 96.8%	*Streptococcus pneumoniae*
11	80 M	1.3×10^7^	*Streptococcus pneumoniae*	*Streptococcus pneumoniae*	51/88, 58.0%	*Streptococcus pneumoniae*
12	70 M	1.3×10^7^	No growth	*Streptococcus intermedius*	57/57, 100%	Oral bacteria
13	80 M	1.9×10^6^	*Moraxella catarrhalis*	*Moraxella catarrhalis*	58/58, 100%	*Moraxella catarrhalis*
14	63 M	4.0×10^6^	*Streptococcus pneumoniae*	*Neisseria mucosa*	14/47, 29.8%	*Streptococcus pneumoniae*
15	73F	1.6×10^5^	*Streptococcus pneumoniae*	*Streptococcus pneumoniae*	78/85, 91.8%	Not analyzed
16	60 M	1.1×10^8^	*Streptococcus pneumoniae*	*Streptococcus pseudopneumoniae*	20/80, 25.0%	Oral bacteria
17	79 M	6.8×10^6^	*Moraxella catarrhalis*	*Moraxella catarrhalis*	57/58, 98.3%	*Escherichia coli*
18	65F	7.6×10^7^	No growth	*Prevotella veroralis*	10/50, 20%	Not analyzed
19	45 M	2.7×10^5^	No growth^#^	*Mycoplasma pneumoniae*	81/84, 96.4%	Oral bacteria
20	80F	2.4×10^6^	*Moraxella catarrhalis, Haemophilus influenzae*	*Moraxella catarrhalis*	50/96, 52.1%	Not analyzed
21	28 M	5.6×10^5^	No growth	*Fusobacterium nucleatum*	51/53, 96.2%	Oral bacteria
22	36F	1.6×10^6^	No growth	*Clostridium* sp.	35/83, 42.2%	Oral bacteria
23	64 M	2.1×10^6^	*Haemophilus influenzae*	*Haemophilus influenzae*	94/94, 100%	*Streptococcus* spp.
24	79 M	2.5×10^8^	*Haemophilus influenzae*	*Haemophilus influenzae*	24/91, 26.4%	Not analyzed
25	91F	1.0×10^8^	*Moraxella catarrhalis*	*Moraxella catarrhalis*	70/96, 72.9%	Not analyzed
26	81F	1.3×10^4^	No growth	*Fusobacterium nucleatum*	44/85, 51.8%	Not analyzed
27	79 M	1.3×10^7^	*Haemophilus influenzae*	*Haemophilus influenzae*	87/95, 91.6%	Not analyzed
28	73F	1.9×10^5^	*Moraxella catarrhalis*	*Moraxella catarrhalis*	69/87, 79.3%	Not analyzed
29	72F	1.9×10^6^	No growth	*Streptococcus pneumoniae*	45/86, 52.3%	*Streptococcus pneumoniae*
30	68 M	1.2×10^7^	*Streptococcus pneumoniae*	*Streptococcus pneumoniae*	92/93, 98.9%	*Streptococcus pneumoniae*
31	23F	4.6×10^6^	No growth^#^	*Mycoplasma pneumoniae*	68/69, 98.6%	Oral bacteria
32	80F	3.4×10^5^	*Pseudomonas cepacia*	*Corynebacterium propinquum*	65/80, 81.3%	Oral bacteria
33	57 M	3.7×10^5^	*Streptococcus pneumoniae, Haemophilus influenzae*	*Streptococcus pneumoniae*	96/96, 100%	Not analyzed
34	69F	2.0×10^6^	*Streptococcus pneumoniae*	*Streptococcus pneumoniae*	62/81, 76.5%	No growth
35	32F	1.9×10^8^	*Staphylococcus aureus*	*Staphylococcus aureus*	63/65, 96.9%	*Staphylococcus aureus*
36	88F	3.1×10^6^	*Haemophilus influenzae*	*Haemophilus influenzae*	69/74, 93.2%	Oral bacteria
37	87 M	1.7×10^6^	*Haemophilus influenzae*	*Streptococcus salivarius*	12/58, 20.7%	*Haemophilus influenzae*
38	64 M	5.6×10^6^	*Prevotella melaninogenica, Streptococcus spp.*	*Haemophilus influenzae*	52/82, 63.4%	Not analyzed
39	71 M	1.9×10^7^	Oral bacteria	*Streptococcus intermedius*	77/77, 100%	Not analyzed
40	73 M	3.1×10^4^	No growth^#^	*Mycoplasma pneumoniae*	93/93, 100%	Oral bacteria
41	16 M	3.4×10^6^	*α-Streptococcus^#^*	*Mycoplasma pneumoniae*	85/88, 96.6%	Not analyzed
42	33 M	7.1×10^5^	*Staphylococcus* sp.	*Neisseria mucosa*	13/61, 21.3%	Not analyzed
43	57F	3.1×10^4^	No growth	*Fusobacterium nucleatum*	31/68, 45.6%	Not analyzed
44	18F	1.4×10^7^	No growth^#^	*Mycoplasma pneumoniae*	88/88, 100%	Not analyzed
45	65F	7.5×10^6^	*Haemophilus* spp. *Streptococcus* spp.	*Haemophilus influenzae*	28/61, 45.9%	Not analyzed
46	63F	2.7×10^5^	*Streptococcus pneumoniae*	*Streptococcus pneumoniae*	37/66, 56.1%	Not analyzed
47	74F	2.3×10^6^	No growth	*Haemophilus influenzae*	75/76, 98.7%	No growth
48	81 M	9.3×10^4^	No growth	*Neisseria perflava*	22/76, 28.9%	Not analyzed
49	28F	1.1×10^5^	*α-Streptococcus^#^*	*Mycoplasma pneumoniae*	70/76, 92.1%	Not analyzed
50	87F	3.7×10^6^	*Haemophilus influenzae*	*Haemophilus influenzae*	23/59, 39.0%	*Haemophilus influenzae*
51	23F	1.5×10^4^	*Actinomyces myeri^#^*	*Mycoplasma pneumoniae*	63/78, 80.8%	*Streptococcus pneumoniae*
52	40F	3.1×10^4^	No growth^#^	*Mycoplasma pneumoniae*	71/82, 86.6%	Oral bacteria
53	57 M	6.8×10^5^	No growth	*Prevotella veroralis*	33/85, 38.8%	Not analyzed
54	84 M	1.5×10^5^	*Streptococcus intermedius*	*Streptococcus intermedius*	58/84, 69.0%	Not analyzed
55	44F	8.6×10^6^	*α-Streptococcus, Neisseria^#^*	*Mycoplasma pneumoniae*	50/81, 61.7%	*Streptococcus* spp.
56	73F	6.2×10^7^	*Staphylococcus aureus, α-Streptococcus*	*Staphylococcus aureus*	48/79, 60.8%	Not analyzed
57	66 M	3.1×10^5^	Oral bacteria	*Veillonella atypica*	17/69, 24.6%	Not analyzed
58	82F	7.6×10^7^	*Haemophilus influenzae, Streptococcus dysgalactiae*	*Haemophilus influenzae*	62/62, 100%	Not analyzed
59	83 M	2.5×10^6^	*Pasteurella multocida*	*Pasteurella multocida*	22/73, 30.1%	Oral bacteria
60	79F	3.7×10^9^	*Haemophilus influenzae*	*Haemophilus influenzae*	93/93, 100%	*Haemophilus influenzae*
61	66 M	3.8×10^7^	Oral bacteria ?	*Prevotella veroralis*	18/77, 23.4%	Not analyzed
62	55 M	1.2×10^6^	*Haemophilus influenzae*	*Haemophilus influenzae*	71/73, 97.3%	Not analyzed
63	32 M	1.0×10^6^	No growth^#^	*Mycoplasma pneumoniae*	41/65, 63.1%	Oral bacteria
64	67F	6.2×10^5^	No growth	*Moraxella catarrhalis*	67/83, 80.7%	*Moraxella catarrhalis*

# Serological assessment of *Mycoplasma pneumoniae* was positive.

### Comparison between the Results of Conventional Cultivation and/or Serological Methods and the Microfloral Analysis of the 16S rRNA Gene

**Figure 1:**
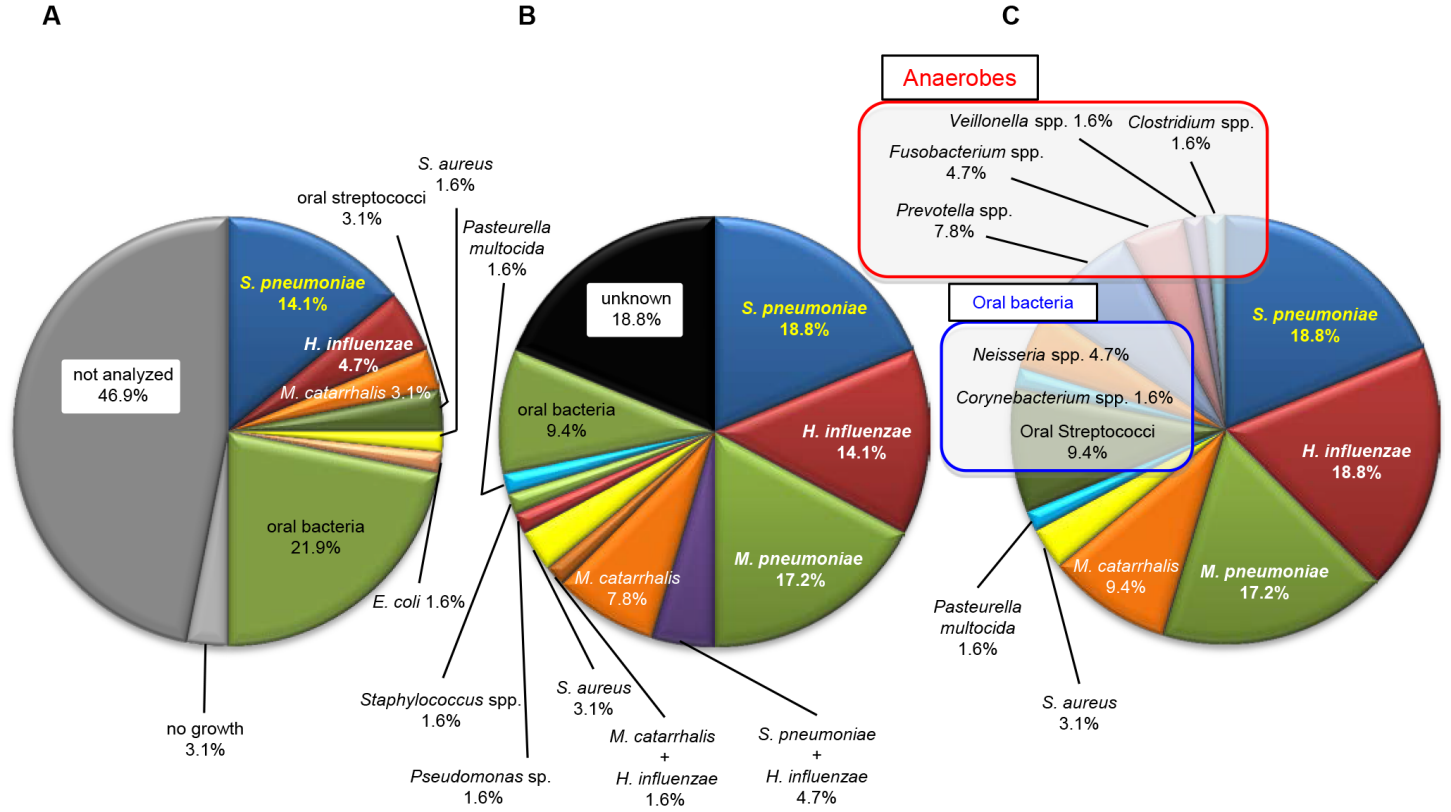
Percentage of detected bacteria by sputum and bronchoalveolar lavage cultivation and the molecular method. The percentage of samples in which bacteria were detected by conventional cultivation of sputum (A), bronchoalveolar lavage (BAL) samples (B) and the molecular method using the 16S rRNA gene (C). The molecular method detected causative bacteria in all BAL samples, and there were considerably higher ratios of oral streptococci and anaerobes detected using the molecular method in comparison to culture methods. “Not analyzed” means that the patients could not produce any sputum for the sputum examination at the time of hospital admission.

Sputum cultivation identified some bacteria in 32 (50.0%) out of the 64 CAP patients, with *S. pneumoniae* being the most commonly detected bacterium (9/64, 14.1%), followed by *H. influenzae* (3/64, 4.7%), *Moraxella catarrhalis* (2/64, 3.1%), and oral streptococci (2/64, 3.1%; Figure 1A). Conventional cultivation of BAL samples and serological methods demonstrated that the most commonly detected pathogen was *S. pneumoniae* (12/64, 18.8%), followed by *M. pneumoniae* (11/64, 17.2%), *H. influenzae* (9/64, 14.1%), and *M. catarrhalis* (5/64, 7.8%). The presence of two or more pathogens was detected in four patients (*S. pneumoniae* and *H. influenzae* in three; *M. catarrhalis* and *H. influenzae* in one) (4/64, 6.3%) (Figure 1B).

The first dominant phylotypes in BAL samples detected by the molecular method (Figure 1C) showed that the most common pathogens responsible for CAP were similarly detected [*S. pneumoniae* (18.8%, 12/64), *H. influenzae* (18.8%, 12/64), *M. pneumoniae* (17.2%, 11/64), and *M. catarrhalis* (9.4%, 6/64)], whereas oral streptococci (9.4%, 6/64), *Neisseria* spp. (4.7%, 3/64), and anaerobes (*Prevotella* spp., *Fusobacterium* spp., *Veillonella* spp., and *Clostridium* sp.) (15.6%, 10/64) were considerably more frequently detected by this molecular method than by the culture method. These sequences have been deposited in GenBank (accession numbers AB787661–AB792640).

In addition, sputum cultivation revealed bacteria other than oral bacteria in 18 patients, and cultured bacteria in 12 out of these 18 sputum samples (66.6%) were consistent with the first dominant bacterial phylotypes detected by the molecular method in BAL samples. [*S. pneumoniae* (7/12), *H. influenzae* (2/12), *M. catarrhalis* (2/12) and *Staphylococcus aureus* (1/12)].

Neither pathogenic organisms nor antigens were detected in 12 (18.8%) out of the 64 CAP patients using the conventional methods, including cultivation, serological examination, and urinary antigen detection. In contrast, anaerobes (*Prevotella* spp. in four; *Fusobacterium* spp. in three; *Clostridium* spp. in one) were highly detected in eight (66.7%) out of these 12 patients by the molecular method.

**Figure 2:**
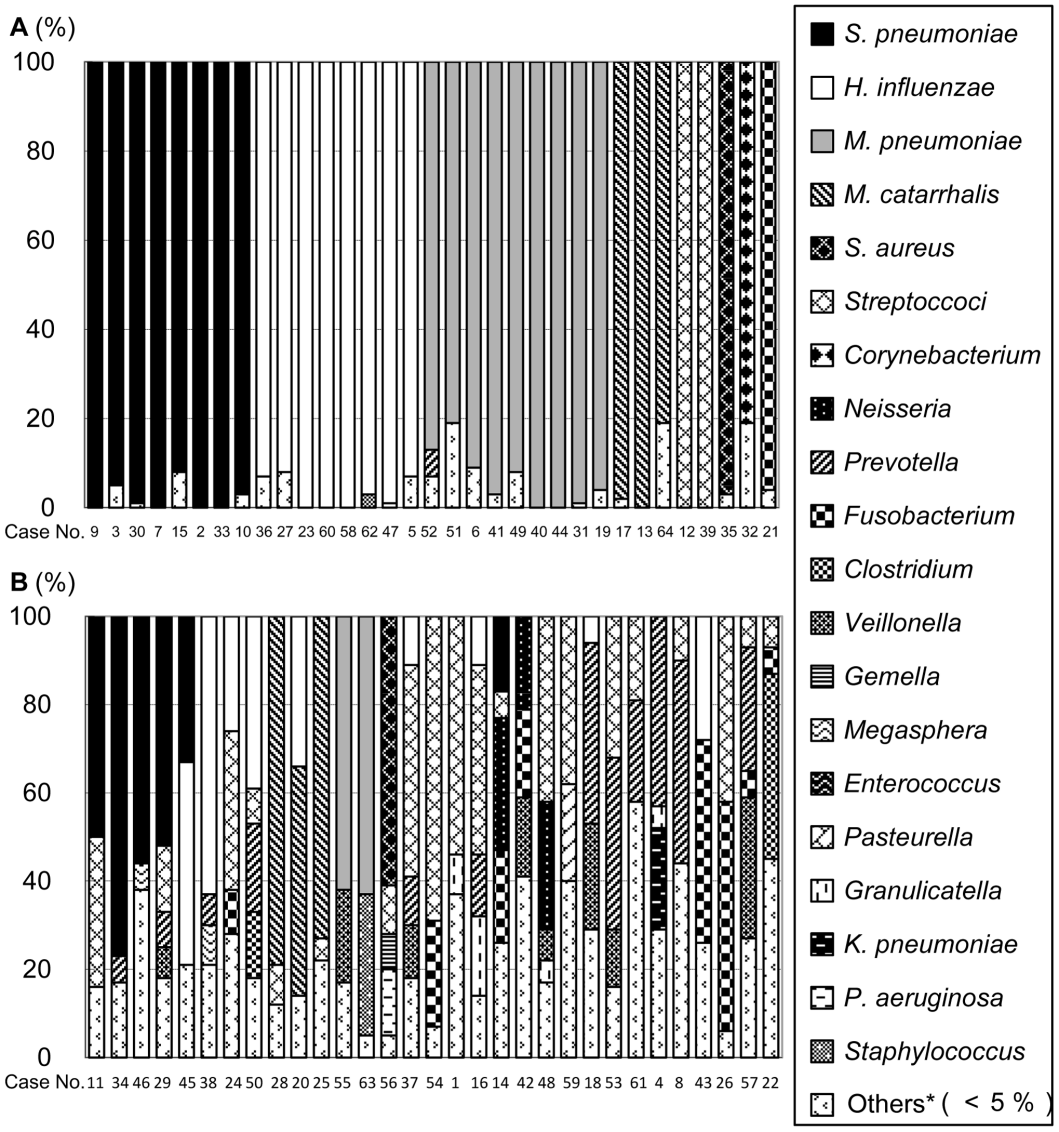
Percentage of detected phylotypes in “monobacterial dominant” and “mixed-bacterial” groups using the molecular method. The percentage of phylotypes in each sample in the 33 patients in the “monobacterial dominant group” (A) and the percentage of phylotypes in each sample in the 31 patients in the “mixed-bacterial group” (B). *Streptococcus pneumoniae*, *Mycoplasma pneumoniae*, *Haemophilus influenzae*, *Moraxella catarrhalis, Staphylococcus aureus, Klebsiella pneumoniae,* and *Pseudomonas aeruginosa* were shown as presumptive species, and the others were shown as presumptive genera. The phylotypes that dominated less than 5% in each library were classified as “Others.”

For descriptive purposes, we defined the “monobacterial dominant group” as the group which included patients in whom the first dominant phylotype comprised over 80% of the detected bacteria, while the other patients were assigned to the “mixed-bacterial group.” According to this definition, 33 patients were categorized as belonging to the monobacterial group, and 31 patients were defined as belonging to the mixed-bacterial group (Figure 2).

Common pathogens of CAP, such as *S. pneumoniae, H. influenzae, M. pneumoniae, M. catarrhalis,* and *S. aureus*, were detected in 29 out of the 33 (87.9%) patients classified into the “monobacterial dominant group,” and oral microbes (6.1%, 2/33) and anaerobes (3.0%, 1/33) were detected in only a few cases. Ten out of the 31 cases (32.3%) classified into the “mixed-bacterial group” had more than 30% of the detected bacterial phylotypes as oral streptococci. (Figure 2).

**Figure 3:**
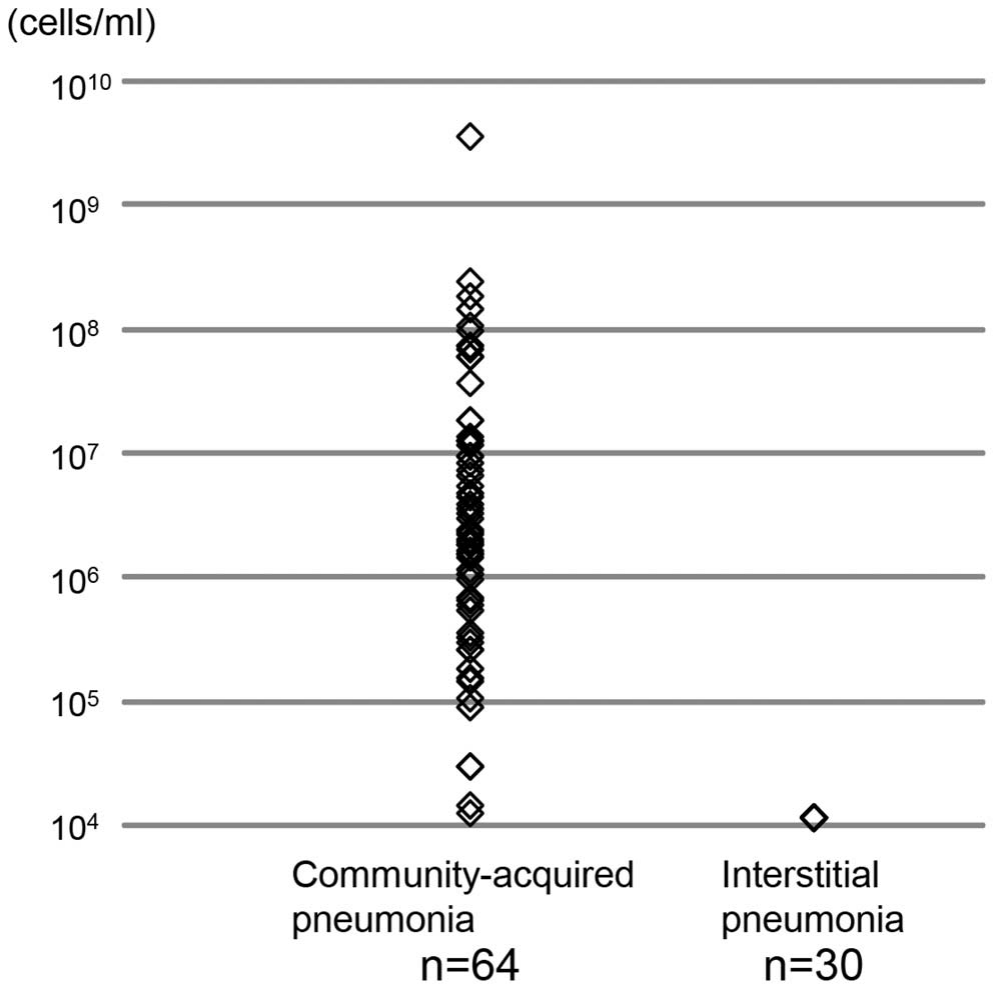
Total number of bacteria in bronchoalveolar lavage samples using epifluorescent microscopic analysis. The total number of bacteria in bronchoalveolar lavage (BAL) samples counted by epifluorescent microscopic evaluations in patients with community-acquired pneumonia and idiopathic interstitial pneumonias. The number of bacteria present in each BAL specimen was counted using an epifluorescent microscopic analysis. The numbers of bacteria in patients with community-acquired pneumonia ranged from 1.3×10^4^ to 3.7×10^9^ (median 2.5×10^6^) cells/ml. On the other hand, all patients with IIPs showed cell counts lower than the detection limit in the epifluorescent microscopic analysis (under 1.3×10^4^ cells/ml).

The BAL specimens obtained from 30 patients with IIPs as representative of noninfectious pulmonary diseases were also evaluated. These specimens showed no bacterial growth and no 16S rRNA gene amplification in any of the subjects using either conventional cultivation methods or the molecular method. In addition, epifluorescent microscopic analyses of these BAL specimens also showed results below the limit of detection (<1.3×10^4^ cells/ml) in all patients with IIPs, in comparison to the results from 64 CAP patients (from 1.3×10^4^ to 3.7×10^9^; median 2.5×10^6^ cells/ml). (Figure 3).

Table 3 shows the ratio of detected bacterial species in each PSI category and each age group. In mild cases, *M. pneumoniae* was the most dominant species, followed by anaerobes, *H. influenzae* and *S. pneumoniae*. *S. pneumoniae*, *M. catarrhalis,* and oral streptococci were predominantly detected in severe cases.

**Table 3:** The first dominant bacterial phylotype in bronchoalveolar lavage fluid.

		PSI								Age							
		Mild		Moderate		Severe		Total		<40y		40–64y		>64y		total	
Pathogen		n	(%)	n	(%)	n	(%)	n	(%)	n	(%)	n	(%)	n	(%)	n	(%)
Aerobes	*Streptococcus pneumoniae*	7	(15.6)	1	(12.5)	4	(36.4)	12	(18.8)	0	(0)	2	(15.4)	10	(25.6)	12	(18.8)
	*Haemophilus* *influenzae*	9	(20.0)	2	(25.0)	1	(9.1)	12	(18.8)	0	(0)	3	(23.1)	9	(23.1)	12	(18.8)
	*Mycoplasma pneumoniae*	11	(24.4)	0	(0)	0	(0)	11	(17.2)	7	(58.3)	3	(23.1)	1	(2.6)	11	(17.2)
	*Moraxella* *Catarrhalis*	3	(6.7)	1	(12.5)	2	(18.2)	6	(9.4)	0	(0)	0	(0)	6	(15.4)	6	(9.4)
	*Staphylococcus* *aureus*	2	(4.4)	0	(0)	0	(0)	2	(3.1)	1	(8.3)	0	(0)	1	(2.6)	2	(3.1)
	*Pasteurella* *Multocida*	0	(0)	0	(0)	1	(9.1)	1	(1.6)	0	(0)	0	(0)	1	(2.6)	1	(1.6)
	*Streptococcus spp.*(except *S.pnuemoniae)*	2	(4.4)	1	(12.5)	3	(27.3)	6	(9.4)	0	(0)	1	(7.7)	5	(12.8)	6	(9.4)
	*Corynebacterium* *spp.*	1	(2.2)	0	(0)	0	(0)	1	(1.6)	0	(0)	0	(0)	1	(2.6)	1	(1.6)
	*Neisseria spp.*	2	(4.4)	1	(12.5)	0	(0)	3	(4.7)	1	(8.3)	1	(7.7)	1	(2.6)	3	(4.7)
Obligatanaerobes	*Prevotella spp.*	5	(11.1)	0	(0)	0	(0)	5	(7.8)	1	(8.3)	2	(15.4)	2	(5.1)	5	(7.8)
	*Fusobacterium* *spp.*	2	(4.4)	1	(12.5)	0	(0)	3	(4.7)	1	(8.3)	1	(7.7)	1	(2.6)	3	(4.7)
	*Veillonella* *spp.*	0	(0)	1	(12.5)	0	(0)	1	(1.6)	0	(0)	0	(0)	1	(2.6)	1	(1.6)
	*Clostridium* *spp.*	1	(2.2)	0	(0)	0	(0)	1	(1.6)	1	(8.3)	0	(0)	0	(0)	1	(1.6)
Total		45	(100)	8	(100)	11	(100)	64	(100)	12	(100)	13	(100)	39	(100)	64	(100)

PSI; pneumonia severity index.

As the first dominant phylotype, *M. pneumoniae* was the most frequently detected phylotype in patients <40 years of age, and *M. pneumoniae*, *H. influenzae* and anaerobes were primarily detected in patients aged 40–64 years. *S. pneumoniae*, *H. influenzae*, *M. catarrhalis,* and oral streptococci were predominantly detected in patients >64 years of age.

## Discussion

In the present study, we analyzed BAL specimens obtained from 64 CAP patients using a clone library analysis of the 16S rRNA gene. To the best of our knowledge, this is the first report to show a higher incidence of anaerobes in CAP patients than was previously believed. Previous reports [3]–[5], [9], [28], [29] have shown that 10–48% microorganisms are etiologically unidentified using traditional culture methods for sputum, in combination with serological and/or specific urinary antigen detection in CAP patients. In contrast to the former reports using cultivation methods, this molecular method could detect bacterial phylotypes in all CAP patients, and the predominant phylotypes were similar to those found in previous reports using traditional cultivation methods [3]–[5]. (Figure 1 and Table 2) In addition, well-known common pathogens of CAP were primarily detected in the “monobacterial dominant group,” whereas most patients in the “mixed-bacterial group” showed multiple bacterial phylotypes including anaerobes and/or oral streptococci.

Using this molecular method, obligate anaerobes such as *Prevotella* spp. and *Fusobacterium* spp. (10/64, 15.6%) and oral streptococci, including *S. intermedius* (6/64, 9.4%), were preferentially detected, especially in CAP patients with unknown etiologies indicated by cultivation-based methods. These results suggest that resident oral streptococci and anaerobes might be the primary bacteria responsible for the unknown causative pathogens of CAP in the previous reports [3]–[5]. These bacteria are generally not considered to be causative pathogens of CAP; however, the higher detection rates of anaerobes in the CAP patients in this study than was previously believed to be present suggest that these bacteria may play important roles in CAP.

We previously applied this molecular method for the etiological evaluation of bacterial pleurisy, and reported that anaerobes were detectable in approximately half of bacterial pleurisies [22]. Moreover, recent molecular studies have shown that anaerobes are frequently detected in patients with stable cystic fibrosis [11].

Oral bacteria (six non-pneumococcal streptococci, three *Neisseria* spp. and one *Corynebacterium* sp.) were detected as the first dominant phylotypes in 10 CAP patients. *S. intermedius* is a member of the *S. anginosus* group, and these bacteria have been reported to range from 1.1% to 3.1% [3]–[5] as causative bacteria in CAP patients. Only a few reports of CAP caused by *S. viridans* (or oral streptococci) or *Neisseria* spp. have been reported [30], although Lambotte *et al.* reported that oral streptococci and *Neisseria* spp. could be causative bacteria in VAP patients [31]. Moreover, 76 (6.8%) out of the 1118 CAP patients showed bacteremic pneumonia and seven (9.2%) of these had positive blood cultures for various non-pneumococcal streptococci in a previous study [32]. All patients with *Neisseria* spp. were relatively immunocompromised in this study; therefore, oral bacteria may preferentially cause CAP in relatively immunocompromised hosts.

Epifluorescent microscopic evaluations in CAP patients and patients with IIPs (Figure 3) demonstrated that the combination of this molecular method and epifluorescent microscopic evaluation detected some bacterial phylotypes only in bacterial infectious diseases. Using this method together with the bronchoscopic method, we were able to avoid any contamination with oral bacteria, which may make it possible to distinguish lower respiratory tract bacterial infections from other noninfectious bronchopulmonary diseases.

There are several limitations associated with this study that should be kept in mind when interpreting the results. First, the universal primers we used could not amplify all of the bacterial 16S rRNA genes, and the sensitivity of the primers was approximately 92% for the bacterial species registered in the Ribosomal Database Project II database. However, the remaining approximately 8% of the bacteria undetectable using these primers does not include any reported human pathogens. Second, the number of clones analyzed in this study was approximately 100 per library, suggesting that this method may not be able to detect bacterial 16S rRNA gene sequences when they are present at very small fractions (less than 1% of each sample). The sequencing depth used in this study is not suitable to detect *Mycobacterium tuberculosis*, which is an important bacterium to assess when obtaining a diagnosis of respiratory disease, even if the bacterium is a minor constituent of clinical specimens.

### Conclusions

We evaluated the causative bacterial species in CAP patients using a microfloral analysis as a cultivation-independent method to detect the presence of the 16S rRNA gene in BAL specimens. The results of our study demonstrate that the incidence of anaerobes and oral bacteria in CAP patients, especially in patients with mild PSI, is higher than previously reported. Therefore, clinicians should consider that anaerobes and oral bacteria are more frequent pathogens than previously believed in CAP patients.
